# Predictors of acute and late diarrhea in the treatment of anal cancer with concurrent chemoradiotherapy

**DOI:** 10.2340/1651-226X.2025.43975

**Published:** 2025-09-15

**Authors:** Katrine Smedegaard Storm, Karen-Lise Garm Spindler, Gitte Fredberg Persson, Claus Behrens, Patrik Sibolt, Sif Homburg, Sofia Spampinato, Camilla Kronborg, Eva Serup-Hansen

**Affiliations:** a Department of Oncology, Copenhagen University Hospital – Herlev and Gentofte, Copenhagen, Denmark; b Department of Oncology, Aarhus University Hospital, Aarhus, Denmark; c Department of Experimental Clinical Oncology, Aarhus University Hospital, Aarhus, Denmark; d Department of Clinical Medicine, Faculty of Health Sciences, University of Copenhagen, Copenhagen, Denmark; e Department of Health Technology, Technical University of Denmark, Kgs Lyngby, Denmark; f Danish Centre for Particle Therapy, Aarhus, Denmark; g Department of Radiotherapy, Erasmus MC Cancer Institute, University Medical Center Rotterdam, Rotterdam, Netherlands; h Department of Clinical Medicine, Aarhus University, Aarhus, Denmark

**Keywords:** Anal canal carcinoma, radiation induced toxicity, contouring methods, NTCP models

## Abstract

**Background and purpose:**

Treatment-related diarrhea is a challenge for patients treated with chemo-radiotherapy (CRT) for anal cancer in a curative setting. This study aims to investigate dosimetric and clinical predictors of acute and late diarrhea for patients treated with CRT or radiotherapy (RT) alone for anal cancer. Additionally, to investigate different bowel contouring methods ability to predict diarrhea.

**Patient/material and methods:**

Patients treated with CRT or RT alone in the prospective, observational DACG-I Plan-A study (2015–2021) were included.

Toxicity endpoints were acute grade ≥2 diarrhea, and late grade ≥1 diarrhea recorded at 1 year after treatment (Common Terminology Criteria of Adverse Events (CTCAE), v4.0).

Bowel volumes were contoured on the planning computed tomography (CT) as bowel cavity, bowel bag, individual bowel loops, and terminal ileum. Dosimetric variables included V_15Gy_, V_30Gy_, and V_45Gy_ for the different bowel volumes. Clinical variables included tumor size, *N*-stage, and chemotherapy regimen. Logistic regression was used to evaluate the association between variables and toxicity.

**Results:**

Of the 290 patients included in this study, 116 (40%) experienced acute grade ≥2 diarrhea, and 56 of 256 (22%) had late grade ≥1 diarrhea. Patients treated with 5-FU/Capecitabine had a threefold higher risk of acute diarrhea compared to those receiving weekly Cisplatin or RT alone (*p* < 0.001). A trend indicating an increased risk of acute grade ≥2 diarrhea for patients with larger bowel volumes receiving radiation was observed. This was most pronounced for bowel bag V_30Gy_ (*p* = 0.09); however, results from the different bowel contouring methods were similar. No parameters were predictive of late diarrhea.

**Interpretation:**

No dosimetric or clinical predictors of late diarrhea were found and only a trend was found between higher dose to bowel and risk of acute diarrhea. Treatment with 5-FU/Capecitabine showed a notable association with acute diarrhea. No contouring method was superior in predicting diarrhea.

## Introduction

Anal cancer (AC) is a relatively rare cancer with an increasing incidence [1]. The primary treatment of non-metastatic AC is chemo-radiotherapy (CRT) [2–5]. Treatment is highly effective but associated with significant acute and late gastrointestinal (GI) toxicity. The incidence of acute diarrhea ranges from 15 to 35% in previous studies [6]. It causes severe morbidity and may lead to treatment delay or modifications, potentially hampering the efficacy. Late toxicity can be debilitating and decrease patients’ quality of life [7].

The dose-response relationship between the risk of treatment-related GI toxicity and bowel radiation dose is well-established from previous studies [8–13]. However, overall translation into robust clinical implementable dose constraints is hampered by inconsistencies in bowel contouring methods and reported outcome measures. Furthermore, results are primarily from retrospective studies of 2D/3D conformal radiotherapy (RT) in smaller study populations of 45–110 patients [8–13]. The focus has been on acute GI toxicity as a composite endpoint and as a result, current dose constraints are based on normal tissue complication probability (NTCP) models for a combined model of acute GI toxicity. A review by Jadon et al. from 2019 exploring dose-response predictors and constraints in late bowel toxicity for RT of several pelvic cancers concluded that studies investigating bowel dose constraints for late toxicity is lacking [14]. In a QUANTEC review from 2010 focusing on pelvic RT, it was suggested that bowel dose constraints applied for acute GI toxicity were also applicable for late toxicity, although they acknowledged the lack of supporting evidence of this [15].

Currently, consensus on bowel volume contouring is conflicting. In the clinic, contouring of individual bowel loops is time-consuming, and often it is preferred to contour the entire peritoneal cavity. Whether it provides a clinical benefit to contour the bowel in more detail or focus on specific parts of the bowel remains unclear. Nevertheless, some studies have recommended contouring the small bowel, large bowel, and bowel loops individually [11–13, 16, 17]. Adding to the complexity, a recent study by Elhaminia et al. found a correlation between radiation dose to the right anterior iliac fossa, where the terminal ileum is located, and risk of treatment-related GI toxicity [18]. This may contribute to diarrhea after RT as it does in other conditions [19].

This study aims to investigate dosimetric and clinical predictors of acute and late treatment-related diarrhea for patients treated with CRT or RT alone for AC. Furthermore, we aimed to determine the optimal bowel contouring model for prediction of treatment-related diarrhea.

## Patients/material and methods

### Patients

From 2015 to 2021 the observational, prospective, DACG-I Plan-A study (*ClinicalTrials.gov Identifier:* NCT05570279) included 314 patients from two centers in Denmark with localized AC eligible for curative CRT or RT alone (trial overview available in supplementary material). Primary endpoints included comparison of prospectively collected acute and late toxicity to dose-volume histograms (DVH) parameters. Patients were treated with Image Guided Radiotherapy (IGRT) with neoadjuvant, concurrent, or no chemotherapy, according to local practice until November 2019 and afterwards according to Danish national guidelines [20]. All patients gave written and oral consent. Patients were staged according to the American Joint Committee on Cancer TNM system version 7 [21]. All patients had a [18F] Fluorodeoxyglucose (FDG) Positron Emission Tomography (PET)-computed tomography (CT) and a Magnetic Resonance Imaging (MRI) done.

### Treatment planning

Radiotherapy planning was based on a CT scan with the patient immobilized in the supine position. No bladder and bowel preparations were required systematically. The treatment planning CT scan was merged with a planning MRI and a diagnostic or planning PET-CT. Primary tumor and pathological lymph nodes (LN) were contoured as Gross Target Volume-Tumor (GTV-T) and Gross Target Volume-Nodes (GTV-N) by a radiologist and radiation oncologist, based on all available imaging and clinical information. The clinical target volume for the primary tumor (CTV-T) was created as a margin from the GTV-T of 10–25 mm to encompass the GTV-T and the entire anal canal. A 10–15 mm CTV margin was added to the GTV-N for the pathological LN. Planning target volume (PTV) extension included a margin of 5–10 mm from the CTV. Daily Cone Beam CTs were done to reduce setup errors for all patients.

The elective volume was contoured according to Australasian GI guidelines and included the mesorectum, presacral space, ischiorectal fossa, bilateral internal and external iliac LN, and bilateral inguinal LN [22]. Modifications and exclusion of elective areas were allowed at the discretion of the prescribing physician depending on disease stage and performance status (PS). Organs at risk (OAR), including the bowel cavity, bladder, femoral heads, os sacrum, penile bulb or vagina, were contoured according to the Radiation Therapy Oncology Group (RTOG) guidelines [23]. Treatment prescriptions included 54–64 Gy to the primary tumor and pathological LN and 48–51.2 Gy to the elective volume in 30–32 fractions, 5 fractions/week. Before 2019, the dose to the tumor was from 60 to 64 Gy according to local practice and Nordic guidelines [24]. After 2019, national guidelines were implemented with small T1 tumors receiving 54 Gy and T2–T4 tumors receiving 60 Gy.

Plan objectives included a 95% coverage of the PTV with 99% of the dose. OAR constraints were defined according to local institutional guidelines until 2018 and included priorities of lowering dose to OAR: (1) bowel cavity, (2) bladder, (3) femoral heads, 4) vagina/penile bulb > testis. From 2018, national guidelines defined constraints to bowel cavity of V_45Gy_ < 300 cm^3^/V_30Gy_ < 600 cm^3^. Intensity-modulated radiotherapy (IMRT) or volumetric-modulated arc therapy (VMAT) techniques were applied.

Different chemotherapy regimens were used during the study period: Regimens were administered according to local practice until 2019 and included: Two cycles of concurrent 5-FU (3,200 mg/m^2^ over 96 h) or Capecitabine (825 mg/m^2^ twice daily) on all radiation days alone or in combination with two cycles of Cisplatin (75 mg/m^2^), or concurrent weekly Cisplatin (40 mg/m^2^), a local practice adapted from the treatment of gynecological cancers. After 2019, national guidelines were implemented and only the regimen with two cycles of concurrent 5-FU (3,200 mg/m^2^ over 96 h) or Capecitabine (825 mg/m^2^ twice daily) on all radiation days was used alone, or in combination with 2 cycles of Cisplatin (75 mg/m^2^). Chemotherapy could be omitted for frail patients or due to comorbidities, in which case the dose could be increased to 64 Gy. Data on treatment interruptions were not available.

### Toxicity scoring

Diarrhea was scored by physicians at mid-treatment, at the end of treatment (EOT), and two to four weeks after treatment, and defined as acute toxicity. Patients in follow-up with no residual or recurrent disease were assessed after 1 year, which was defined as late toxicity. Scoring was done with the Common Terminology Criteria of Adverse Events (CTCAE) version 4.

### Contouring of bowel

**Figure 1:**
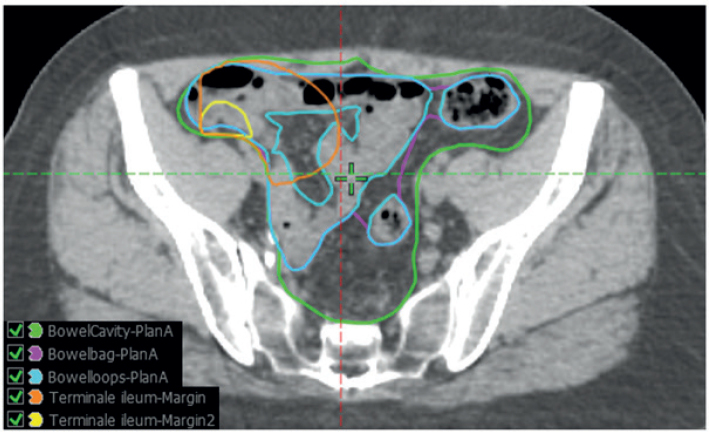
The five different bowel contouring methods visualized in transversal, sagittal and coronal planes.

The bowel cavity was contoured for treatment planning and was verified for post-hoc analysis and corrected if necessary to ensure correct and comparable contouring according to guidelines. Other bowel contours were contoured post-hoc on the planning CT by two clinical oncologists (KAS and SH) in the eclipse treatment planning system (TPS, Varian, a Siemens Healthineers Company). Contours included (Figure 1):

1. ***Bowel cavity;*** including the peritoneal cavity from the abdominal wall excluding the bladder, uterus, prostate, rectum, and large vessels from the lower limit of L4 vertebrae to the last visible bowel loop as per RTOG guidelines [23].
2. ***Bowel loops;*** including all individual small and large bowel loops from the lower limit of L4 vertebrae to the last visible bowel loop.
3. ***Bowel bag;*** Only the volume of the peritoneal cavity, which includes visible small and large bowel loops, is contoured as an ‘envelope’ around the bowel loops as per Devisetty et al. [12].
4. ***Terminal ileum;*** Defined along two different not previously defined approaches. The central point of the ileocecal valve was identified on a single axial slice, and subsequently, an anisotropic margin was added and shaped to the peritoneal cavity. For the first approach (Terminal ileum 1), the margin applied was 10 mm in the cranial direction, 70 mm in the caudal direction, 50 mm in the medial direction, 30 mm in the anterior direction, and 20 mm in the posterior direction, which was assumed to encompass the entire terminal ileum. For the second approach (Terminal ileum 2), a more uniform margin of 10 mm in the cranial direction and 30 mm in the anterior, posterior, medial, and caudal directions was added.

The ileocecal valve was visible for all but two patients. In case of doubt, a consensus was reached between at least two of four oncologists (KAS, SH, CK, ESH).

### Statistical analysis

Two toxicity endpoints were examined: acute grade ≥2 diarrhea (defined as the highest score of mid-treatment, EOT, and 4 weeks after EOT) and late grade ≥1 diarrhea. Patients were included for analysis of acute diarrhea if they had at least one acute toxicity score registered. Only recurrence-free patients with toxicity registered at 1 year were included for analysis of late diarrhea.

Dosimetric data included the absolute volume of bowel receiving 15, 30, and 45 Gy, respectively (V_15Gy_, V_30Gy_, and V_45Gy_). These specific parameters were chosen due to findings in previous studies of pelvic RT and their current use as dose constraints in treatment planning of AC [25–27]. In contrast, as no specific dose-volume parameters exist for reporting of dose to the terminal ileum, several DVH parameters were evaluated to facilitate an exploratory analysis: V*x* Gy where *x* ranged from 5 to 45 Gy with 5 Gy intervals. Data were extracted from the treatment plan DVH for the different bowel contouring methods.

The distributions of dosimetric values for patients with or without toxicities were compared using the Wilcoxon rank sum test. Univariate logistic regression was used for evaluation of the association of clinical parameters and toxicity. Baseline parameters that were considered clinically relevant included tumor size (T1–T4), *N*-stage (N0/N1–3), gender (female/male), Performance Status (0–3), radiation dose (60/48 or 49.5 Gy/30 F, 64/51.2 Gy/32 F and 54/48 Gy/30 F or tumor only), and chemotherapy regimen (No Chemotherapy, Weekly Cisplatin and 5-FU/Capecitabine ± Cisplatin). A *p*-value of 0.1 was used for selection of variables for multivariate regression analysis. The dosimetric variables considered clinically relevant and showing the most association with toxicity endpoints underwent further investigation with multivariable logistic regression analysis adjusting for clinically significant parameters creating an NTCP-model, using the following equation:


$$p=\frac{1}{1+{\text{e}}^{-\left(\text{β}0+\text{β}1\text{X}+\text{β}2\text{X}+\text{β}3\text{X}\ldots \right)}}$$

Goodness-of-fit was assessed using the Hosmer-Lemeshow test, AUC, and calibration plot (plots in supplementary). A *p*-value of 0.05 was considered significant.

The study was conducted as an exploratory analysis. Multiple testing was used, which increases the risks of overinterpreting findings. However, to avoid the risk of Type II errors and potentially missing significant predictors, we did not correct for multiple testing, and a significance level of 0.1 was used to select parameters for the multivariate analysis.

All statistical analyses were performed using R studio (version 4.2.2, RStudio Team, Boston, MA, USA).

## Results

Of the 310 patients that were included in DACG-I Plan-A from 2015 to 2021, 290 patients had at least one toxicity registration for acute diarrhea and were included for analysis. Of those, 281 (97%) had at least two registered. One-year toxicity score was registered for 256 of the 290 patients (88%) and was included in the analysis of late diarrhea (a flow diagram can be found in supplementary material). Patient demographics, disease, and treatment characteristics can be found in Table 1. Of the 290 patients included for analysis, 178 patients (61%) received a concurrent chemotherapy regimen including 5-FU or Capecitabine, 42 patients (15%) received weekly Cisplatin, and 69 patients (24%) were treated with RT only. The combination of 60/48 Gy in 30 Fx and Cisplatin with 5-FU or Capecitabin was most frequently used, with 40% of patients receiving this treatment (table in supplementary).

**Table 1:** Baseline characteristics for all patients including treatment details, demographics, and acute and late diarrhea score.

Patient, disease, and treatment characteristics	All patients (*n* = 290)
**Age** Median [Range], years	64 [26,87]
**Gender** No. (%)	
Male	76 (26)
Female	214 (74)
**Performance status** No. (%)	
0	216 (74)
1	63 (22)
2	11 (4)
**P16-status** No. (%)	
Positive	238 (82)
Negative	23 (8)
Unknown	29 (10)
***T*-stage** No. (%)	
T1	47 (16)
T2	148 (51)
T3	45 (16)
T4	50 (17)
***N*-stage** No. (%)	
N0	192 (66)
N1	41 (14)
N2	39 (13)
N3	18 (6)
***M*-stage** No. (%)	
M0	281 (97)
M1	9 (3)
**Tumor size** Median [range], mm	35 [0.150]
**Tumor location** No. (%)	
External	38 (13)
Anal canal	227 (78)
Anal verge	104 (36)
Rectum	54 (19)
**Radiation dose** No. (%)	
60/49.5 Gy/30 F	175 (60)
64/51.2 Gy/32 F	73 (25)
60/48 Gy/30 F 54/48 Gy/30 F	24 (8) 5 (2)
54 or 60 Gy/30 F (tumor only)	9 (3)
48–54 Gy/27 F (tumor only)	4 (1)
**Neoadjuvant chemotherapy** No. (%)	
Yes	14 (5)
No	276 (95)
**Chemotherapy** No. (%)	
Cisplatin+5-FU/Capecitabine	142 (49)
5-FU/Capecitabine	36 (12)
Weekly Cisplatin	43 (15)
No Chemotherapy	69 (24)
Acute peak toxicity score* No. (%)	
0	71 (24)
1	103 (36)
2	75 (26)
3	40 (14)
4	1 (0)
Late toxicity score** No. (%)	
0	200 (69)
1	41 (14)
2	11 (4)
3	4 (1)
Unknown	34 (12)

* Acute diarrhea defined as highest score at mid-treatment, end of treatment, and 2 to 4 weeks after treatment.

** Late diarrhea as recorded at 1 year FU.

Acute diarrhea grade ≥ 2 was observed in 116 out of 290 (40%) patients, 40 patients (14%) had grade 3 diarrhea, and only one patient (0.3%) experienced grade 4. Grade ≥ 1 late diarrhea was seen in 56 out of 256 patients (22%) with 11 patients (4%) experiencing grade 2 diarrhea and four (1.5%) patients experiencing grade 3.

### Dosimetric variables and acute diarrhea

Treatment plans complied with bowel cavity V_45Gy_ and V_30Gy_ dose constraints in 43% and 52% of the dose plans, respectively.

Median values for the dosimetric parameters were generally higher for patients with acute grade ≥ 2 diarrhea compared to the group of patients with grade 0–1 (Table 2). However, comparing the distributions of the clinically relevant dose volume parameters between the two groups revealed no large differences for any bowel contours applied. The greatest trend was found for bowel bag V_30Gy_ (*p* = 0.09) with a median volume Interquartile range (IQR) of 368 cm^3^ [191;523] for patients with grade 1 or no toxicity and 385 cm^3^ [237;582] for patients with grade ≥2 acute toxicity. However, other contouring methods were comparable, such as bowel cavity V_30Gy_ (*p* = 0.11) with a median volume [IQR] of 566 cm^3^ [350;785] for patients with grade 1 or no toxicity and 614 cm^3^ [393;809] for patients with grade ≥2 acute toxicity. The dosimetric parameters for the terminal ileum contours were similar for the two groups.

**Table 2:** Dosimetric variables and the correlation with acute diarrhea grade 0–1 and grade ≥ 2 using the Wilcoxon rank sum test. A p-value of 0.1 or less is highlighted in bold

Acute toxicity (290)			
Median [IQR]	Grade 0–1 (*n* = 174)	Grade ≥ 2 (*n* = 116)	*p*
**Bowel cavity**			
V15Gy (cm^3^)	925.6 [606.1;1192.8]	943.5 [669.0;1205.5]	0.16
V30Gy (cm^3^)	565.8 [349.5;785.1]	614.3 [393.1;809.1]	0.11
V45Gy (cm^3^)	322.3 [166.9;458.8]	347.8 [217.3;460.1]	0.23
**Bowel bag**			
V15Gy (cm^3^) V30Gy (cm^3^) V45Gy (cm^3^)	613.8 [361.9;822.0] 367.5 [191.1;522.9] 157.1 [60.5;289.3]	631.6 [439.8;862.0] 385.4 [236.7;582.1] 194.8 [94.0;293.1]	0.22 **0.09** 0.21
**Bowel loops**			
V15Gy (cm^3^) V30Gy (cm^3^) V45Gy (cm^3^)	433 [257.8;622.4] 241.6 [120.7;393.6] 120.5 [47.5;227.5]	458.2 [296.8;596.8] 277.4 [156.5;393.3] 136.9 [63.3;226.7]	0.51 0.31 0.43
**Terminal ileum 1**			
V10Gy (cm^3^)	106.8 [19.4;194.5]	117.8 [33.25;163.9]	0.84
V15Gy (cm^3^)	88.6 [7.3;152.8]	97.8 [21.8;149.5]	0.72
V20Gy (cm^3^)	75.4 [1.9;134.8]	84.6 [15.5;128:8]	0.81
V25Gy (cm^3^)	59.0 [0.5;107.7]	59.0 [7.3;114.1]	0.71
V30Gy (cm^3^)	41.9 [0.4;81.8]	45.5 [1.2;90.2]	0.53
V35Gy (cm^3^)	26.4 [0;62.5]	24.9 [0.1;69.6]	0.44
V40Gy (cm^3^)	18.55 [0;46.6]	18.1 [0;52.8]	0.45
**Terminal ileum 2**			
V10Gy (cm^3^)	17.6 [0;54.3]	27 [0;58]	0.75
V15Gy (cm^3^)	7.6 [0;48.4]	17.2 [0;51.3]	0.68
V20Gy (cm^3^)	3.3 [0;39.4]	8.4 [0;36.5]	0.86
V25Gy (cm^3^)	0.7 [0;28.2]	4.6 [0;28.3]	0.93
V30Gy (cm^3^)	0.1 [0;18.1]	1.1 [0;17.8]	0.83

### Dosimetric variables and late diarrhea

No dosimetric parameters were clearly distinguishable between the late toxicity groups for any contouring methods (Supplementary Materials Table 5), rendering no significant prediction of toxicity. and further modeling of late diarrhea dosimetric predictors was omitted.

### Clinical parameters

Univariate logistic regression analysis of clinical parameters showed a significantly increased risk of grade ≥2 acute diarrhea for patients treated with a chemotherapy regimen that included 5-FU or Capecitabine compared to patients treated without chemotherapy or with weekly Cisplatin (OR 2.92 [1.57;5.44], *p* < 0.001) (Table 3). For acute toxicity, a higher *T*-stage also increased risk of toxicity, for T2 tumors (OR 1.83 [0.89;3.76], *p* = 0.09), T3 tumors (OR 2.29 [0.96;5.45], *p* = 0.06), and T4 tumors (OR 1.89 [0.81;4.43], *p* = 0.14) although not significant for T4 tumors and with wide CI intervals. Patients with positive LN also showed an increased risk of toxicity in univariate analysis (OR 1.54 [0.94;2.53], *p* = 0.09).

**Table 3:** Logistic regression of clinical and selected dosimetric predictors of acute and late diarrhea. Odds ratio with 95% confidence intervals (CI) and p-value. A p-value of 0.1 or less for univariate analysis and a p-value of 0.05 or less for multivariate analysis is highlighted in bold. *Odds ratio per 10 cm_3_ increase.

Univariate analysis	Acute diarrhea Grade ≥ 2		Late diarrhea Grade ≥ 1	
	OR [95% CI]	*p*	OR [95% CI]	*p*
***T*-stage**				
T1	reference		Reference	
T2	**1.83 [0.89;3.76]**	**0.09**	1.03 [0.47;2.26]	0.94
T3	**2.29 [0.96;5.45]**	**0.06**	0.40 [0.11;1.39]	0.15
T4	1.89 [0.81;4.43]	0.14	0.55 [0.19;0.59]	0.27
***N*-stage**				
N0	reference		Reference	
N1–3	**1.54 [0.94;2.53]**	**0.09**	**0.54 [0.27;1.07]**	**0.08**
**Tumor size (mm)**	1.08 [0.96;1.21]	0.20	**0.84 [0.70;1.00]**	**0.05**
**ECOG Performance status**				
PS 0	Reference		Reference	
PS 1	1.04 [0.59;1.84]	0.89	**0.37 [0.14;0.98]**	**0.05**
PS 2	0.56 [0.14;2.15]	0.40	1.90 [0.44;8.24]	0.39
**Male gender**	0.97 [0.57;1.66]	0.91	0.84 [0.42;1.68]	0.62
**Age at diagnosis (years)**	1.00 [0.98;1.02]	0.94	1.00 [0.97;1.03]	0.89
**Radiation dose (Gy)**				
60/48 or 49.5 Gy/30 F	Reference		Reference	
64/51.2 Gy/32 F	**0.45 [0.25;0.80]**	**0.007**	1.53 [0.78;2.99]	0.21
54/48 Gy/30 F or tumor only	**0.07 [0.01; 0.05]**	**0.009**	0.68 [0.08;5.80]	0.72
**Chemotherapy**				
No Chemotherapy	Reference		Reference	
Weekly Cisplatin	1.18 [0.50;2.81]	0.70	2.01 [0.72;5.57]	0.18
5-FU/Capecitabine ± Cisplatin	**2.92 [1.57;5.44]**	**<0.001**	1.52 [0.68;3.39]	0.30
**Multivariate – late toxicity**				
tumor_size			0.88 [0.73;1.07]	0.20
N0			Reference	
N1–3			0.70 [0.33;1.50]	0.36
PS 0			Reference	
PS 1			0.47 [0.17;1.29]	0.14
PS 2			2.15 [0.48;9.57]	0.31
**Multivariate – acute toxicity**				
Bowelbag V_30Gy_	1.0 [1.00;1.00]	0.21		
No Chemotherapy	Reference			
Weekly Cisplatin	1.26 [0.45;3.51]	0.65		
5-FU/Capecitabine ± Cisplatin	**2.52 [1.23;5.13]**	**0.01**		
**tumor_size**	0.98 [0.80;1.20]	0.83		
**T1**	Reference			
**T2**	1.10 [0.45;2.68]	0.84		
**T3**	1.22 [0.30;4.97]	0.78		
**T4**	0.95 [0.27;3.40]	0.94		
**N0**	Reference			
**N1–3**	1.12 [0.63;2.01]	0.69		
**60/48 or 49.5 Gy/30 F**	Reference			
64/51.2 Gy/32 F	0.68 [0.29;1.58]	0.37		
54/48 Gy/30 F or tumor only	0.12 [0.01;1.03]	0.06		
**Multivariate analysis**				
Bowel bag V30 Gy	[1.00;1.02]	0.08		
No Chemotherapy	Reference			
Weekly Cisplatin	1.16 [0.49;2.76]	0.74		
5-FU/Capecitabine ± Cisplatin	**2.95 [1.58;5.53]**	**>0.001**		

PS: performance status.

Patients treated with an RT dose of 60 Gy to tumor and with irradiation of elective areas had a higher risk of acute diarrhea compared to patients treated with 64 Gy (OR 0.45 [0.25;0.80], *p* = 0.007), or lower dose of 54/48 Gy or tumor only (0.07 [0.01; 0.05], *p* = 0.009). In multivariate regression analysis including these parameters, only patients treated with a chemotherapy regimen that included 5-FU or Capecitabine had an increased risk of grade ≥2 acute diarrhea (2.52 [1.23;5.13], *p* = 0.01).

In univariate analysis of clinical parameters and late diarrhea, a lower risk of late diarrhea grade ≥ 1 was seen for patients with lymph node involvement (0.54 [0.27;1.07], *p* = 0.08), larger tumor size (0.84 [0.70;1.00], *p* = 0.05), and PS 1 compared to PS 0 (0.37 [0.14;0.98], *p* = 0.05). However, in multivariate regression analysis, there were no significant parameters (Table 3).

### Normal tissue complication probability model

**Figure 2:**
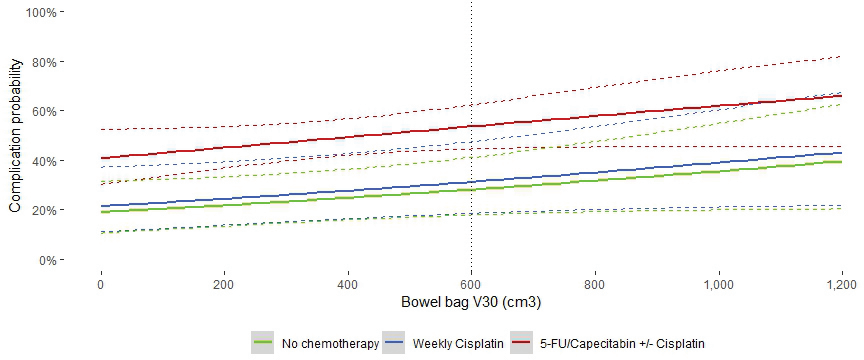
Logistic NTCP model for acute grade ≥2 diarrhea and bowel bag V_30GY_. The corresponding 95% confidence intervals are illustrated with bands. The dotted vertical line represents the current dose constraint.

Further analysis of acute toxicity and dosimetric predictors used multivariate logistic regression for NTCP modeling, focusing on bowel bag V_30Gy_ (Table 3). As described above, among the dosimetric variables, bowel bag V_30Gy_ showed the best separation between the two groups (*p* = 0.09) for acute diarrhea and was chosen as the best-performing parameter for NTCP modeling. The model was adjusted for chemotherapy regimen divided into 5-FU or Capecitabine ± Cisplatin, weekly Cisplatin, or no chemotherapy (Figure 2) based on results from multivariate regression analysis with the following equation: logit(p) = −1.4527+0.0008564×bowelbag_V30_+0.1494× I (weekly cisplatin) +1.0833×I (5-FU/Capecitabine ± Cisplatin).

Goodness of fit analysis with the Hosmer-Lemeshow test showed a reasonable fit with a p-value of 0.11. AUC [95% CI] for the NTCP model was 0.646 [ 0.582 - 0.71].

Goodness of fit AUC analysis and calibration plot are shown in the supplementary.

## Discussion and conclusion

Including almost 300 patients with AC, we present the largest prospective study on predictors of treatment-induced acute and late diarrhea. One important finding was that none of the bowel contours were superior in predicting acute or late diarrhea. Although bowel bag V_30Gy_ was the best-performing dosimetric parameter, the results were very similar between the different contouring methods, and no strong predictor of acute grade ≥ 2 diarrhea was seen.

We found a rate of acute grade ≥2 diarrhea of 40%, which is slightly higher than previously reported rates of 35% in a study from 2018 by Olsen et al. [13] or 32.2% in a prospective study by Lukovic et al. from 2022 [16]. During the study period, various treatment combinations were used, and could potentially bias the results, particularly concerning the different chemotherapy regimens used. Nevertheless, the extensive size of the study cohort offers a chance to investigate the effects of different regimens, including weekly Cisplatin. While weekly Cisplatin is no longer part of AC treatment, it remains a component in the therapy for cervical cancer [28] and showed potential in a previous study, even though it is not a standard in the treatment of AC [29]. The analysis included 14 patients (5%) who received neoadjuvant chemotherapy. It was expected that these patients might have had more chemotherapy-induced toxicity, potentially biasing the outcome. However, a sub-analysis revealed no higher baseline or acute toxicity in this group.

No prior studies, using modern RT techniques, have included such a large group of patients receiving RT alone. We observed an almost three times higher risk of acute grade ≥ 2 diarrhea in patients treated with concurrent 5-FU or Capecitabine and RT compared to RT alone. International guidelines currently recommend mitomycin C in combination with 5-FU or capecitabine as standard concurrent chemotherapy [30]. In the present study, chemotherapy regimens included cisplatin rather than mitomycin C, in accordance with national Danish treatment guidelines [20]. While this difference may limit direct comparability with previous studies, the randomized controlled trials ACT-II and RTOG 9811 found no significant differences in toxicity between mitomycin- and cisplatin-based CRT in the treatment of AC [31, 32]. These findings suggest that the results of this study are also applicable to regimens combining 5-FU with mitomycin C. Concurrent chemotherapy was implemented after the randomized phase III trials, ACT I [33, 34] and EORTC 110 [4], that demonstrated increased overall survival when adding chemotherapy to radiation. Both studies only included a small number of patients with T1 tumors. Whether RT alone suffices for small tumors is still debated; for example, Deniaud-Alexandre et al. reported a 96% complete response rate for T1 tumors and 87% for T2 tumors with RT alone [35]. For the larger and more advanced tumors, there is no doubt that the addition of chemotherapy is beneficial. However, for patients where acute diarrhea hampers the chance of treatment completion or causes treatment delays, it may be better to reduce the chemotherapy dose than to reduce the radiation dose or the target volumes. Data on treatment interruptions were not available in this study [8, 11, 12, 17].

Previous studies have found a correlation of bowel bag V_30Gy_ with GI-toxicity. Devisetty et al. found a significant correlation between bowel bag V_30Gy_ and patients experiencing grade ≥ 2B acute GI toxicity (OR 1.47, 95% CI [1.02–2.12]), suggesting a constraint of bowel bag V_30Gy_ < 450 cm^3^ [12]. Nilsson et al reported retrospective data on acute grade ≥ 3 GI toxicity on 114 patients and found a significant correlation with bowel bag V_30Gy_ [11]. The findings from these earlier studies imply that bowel bag V_30Gy_ serves as a relevant measure in clinical settings. However, these prior studies included fewer patients, and one was a retrospective study. Our focus on a single toxicity endpoint instead of overall GI toxicity could also explain why our study found no strong correlation. We chose to focus on linking bowel volumes to acute diarrhea and not overall GI toxicity, recognizing that other GI toxicities are not necessarily only linked to irradiation of bowel. GI toxicity encompasses symptoms such as nausea, vomiting, fecal incontinence, urge, and proctitis, among others. We examined the endpoint of grade ≥ 2 and not grade 3 ≥ diarrhea. 41 patients (14%) experienced grade 3 or 4 diarrhea, and further analysis did not show an association with dose to bowel, perhaps due to the low number of events. Furthermore, since this study was based on exploratory analyses, the results may be prone to type I and type II errors, potentially leading to overestimation or omission of significant findings.

We explored a low dose (V_15Gy_), medium dose (V_30Gy_), and high dose (V_45Gy_). The high dose of 45 Gy is close to the dose given to the elective areas of 48 or 49.5 Gy. Due to the steep dose gradient in that area, there can be large variabilities and uncertainty in actual dose delivered when based solely on the reference scan and not daily CBCTs. Therefore, investigating the low or medium dose volumes may give a more robust dose-response relationships. Bowel bag V_30Gy_ was chosen to demonstrate the trend of increasing risk of toxicity with increased dose because of its current use as constraint and optimization parameter in dose planning and the increased risk of acute diarrhea when treated concurrently with a 5-FU/Capecitabine chemotherapy regimen. In the presented NTCP model for bowel bag V_30Gy_, no relevant cut-off point for dose constraint was found but rather a continuous minor increased risk. The model can thus be used for clinical risk estimation [36]. Patient-reported outcome toxicity (PRO) is suggested as a superior toxicity measurement for NTCP modeling. In a previous study from the PLAN-A cohort, Kronborg et al. found that interrater agreement between EORTC-PRO and CTCAE was fair for items related to diarrhea, although grading was higher for EORTC-PRO. This discrepancy suggests that using PRO instead of CTCAE as the toxicity endpoint could lead to different outcomes [37].

Our findings indicate that a more detailed contouring method does not increase the ability to predict acute diarrhea. A prospective study where plans were optimized based on the bowel contours may yield a different result, but no such data exist. Therefore, we suggest using the simplest, less time-consuming, contouring method for clinical evaluation in everyday practice. Contouring of small bowel separately was not feasible in this study, as the planning CT was done without contrast; evaluation of this contour is therefore beyond the scope of this work.

Our data did not support the correlation between dose to the terminal ileum and acute or late diarrhea as suggested by Elhaminia et al. [18]. Defining the terminal ileum on a CT without contrast is difficult, and the two different anisotropic margins that were created from the ileocecal valve to ensure inclusion of the entire terminal ileum were exploratory. Whether the lacking correlation was due to a true lack of correlation or insufficient contouring of the terminal ileum remains unclear. The study by Elhaminia et al. also had bowel urgency as the primary outcome, which may explain the lack of correlation with diarrhea in this study. Furthermore, their dataset included patients with anal, rectal, and gynecological cancers, many of whom were treated with conventional 3D RT technique

Lastly, none of the analyzed dosimetric or clinical variables were predictors of 1-year observed late diarrhea. This may change with longer follow-up in the trial, and three- and 5-year late toxicity will be available in 2026. The CTCAE definition for grade 1 diarrhea (an increase of fewer than 4 stools per day from baseline) is less robust than for grade 2 or higher. However, the number of patients experiencing grade 2 late diarrhea in this cohort was too low to allow for meaningful statistical analysis.

## Conclusion

To conclude, no dosimetric or clinical predictors of late diarrhea were found, and only a trend was found between higher dose to bowel and risk of acute diarrhea. Treatment with 5-FU/Capecitabine showed a notable association with acute diarrhea. No contouring method was superior in predicting diarrhea.

## Supplementary Material



## Data Availability

Research data are not available at this time due to strict GDPR regulations.
