# Supplementary material for: Predictors of acute and late diarrhea in the treatment of anal cancer with concurrent chemoradiotherapy
Source: Acta Oncol. 2025 Sep 15;64:43975. doi: 10.2340/1651-226X.2025.43975 (PMC12449700; doi:10.2340/1651-226X.2025.43975)
Supplement: Supplementary file 1 [file AO-64-43975-s1.pdf]

Supplementary material has been published as submitted. It has not been copyedited, or typeset by Acta Oncologica

Supplementary.

Flowchart of patient inclusion and missing data

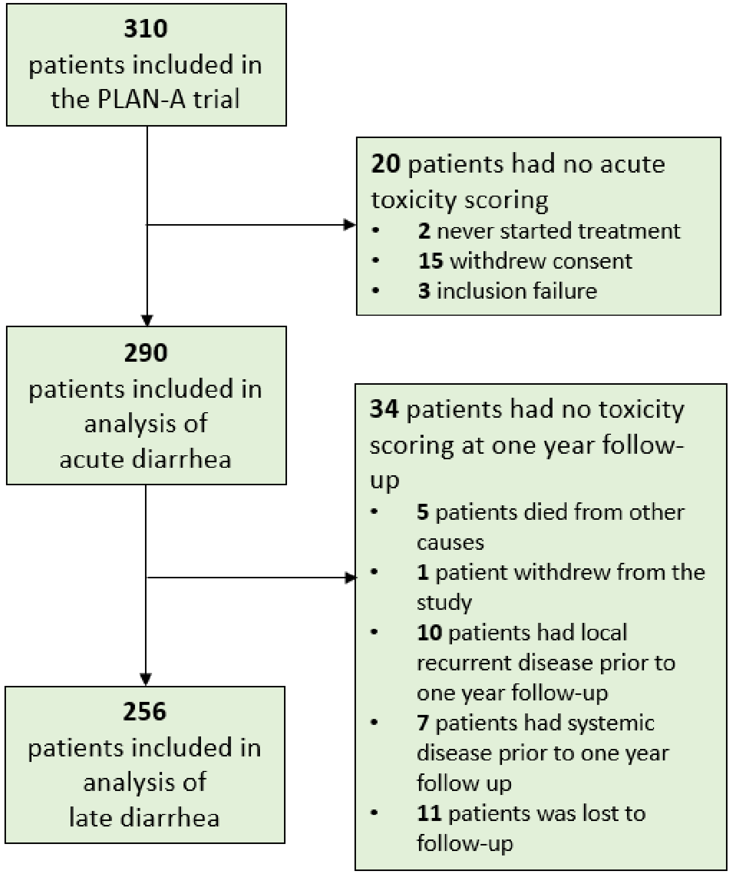

PLAN-A study overview

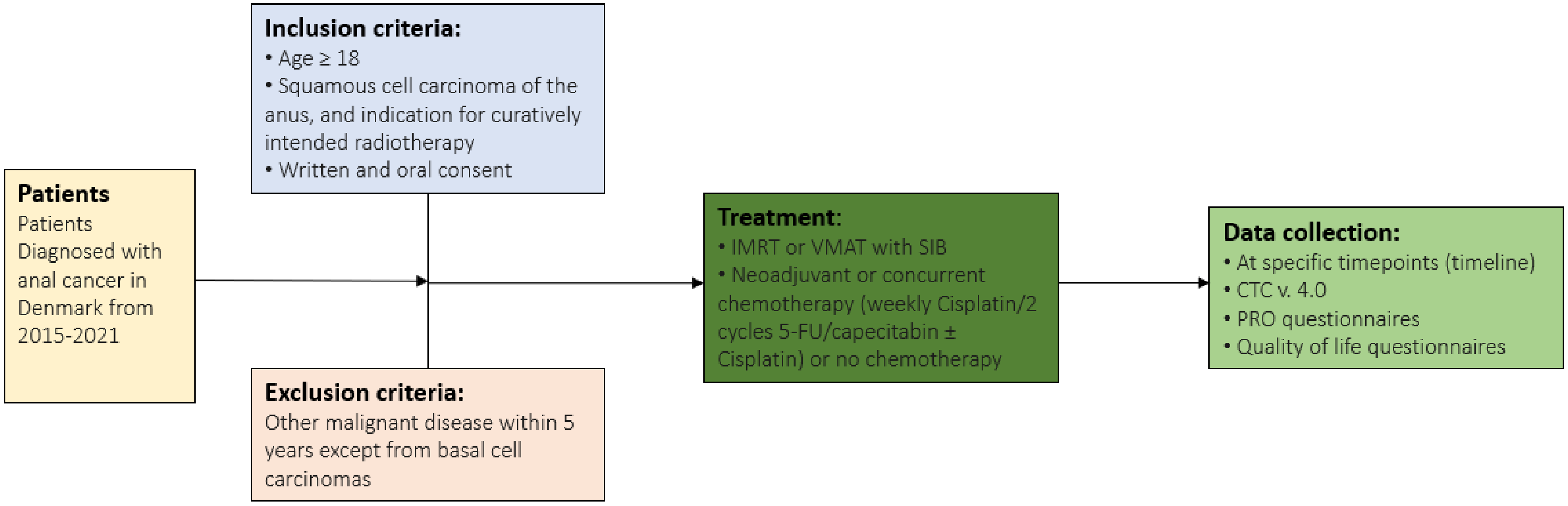

Patient timeline

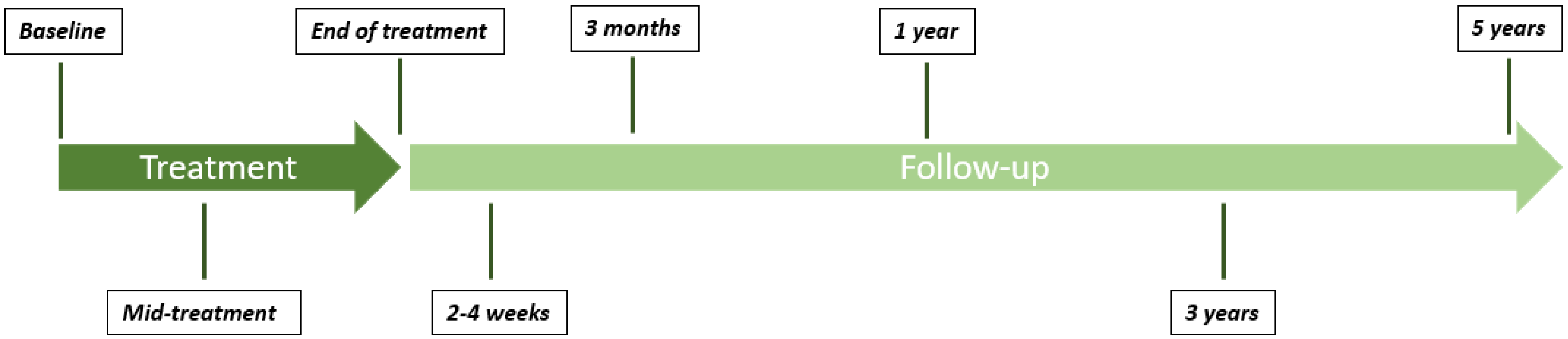

	**Cisplatin+5-FU/Capecitabine (n=142)**	**5-FU/Capecitabine only (n=36)**	**Weekly Cisplatin (n=43)**	**No Chemotherapy (n=69)**
**60/48 or 49.5 Gy/30 F (n=199)**	132 (46)	28 (10)	3 (1)	36 (12)
**64/51.2 Gy/32 F (n=73)**	6 (2)	3 (1)	38 (13)	26 (9)
**54/48 Gy/30 F (n=5)**	2 (0.7)	1 (0.3)	1 (0.3)	1 (0.3)
**54 or 60 Gy/30 F (Tumor only) (n=9)**	2 (0.7)	1 (0.3)	1 (0.3)	5 (2)
**48-54 Gy/27 F (Tumor only) (n=4)**	0	3 (1)	0	1 (0.3)

Table 4. The different radiotherapy and chemotherapy regimens during the study period, and number (percentage) of patients receiving the different combinations.

Table 5. Dosimetric variables and the correlation with late diarrhea grade 0 and 1 or higher using Wilcoxon rank test

**Median** [IQR] (n=256)	**Grade 0** (n= 200)	**Grade ≥ 1** (n=56)	**P-value**
**Bowel cavity** (cm^3)			
V15Gy	920.5 [601.0;1174.1]	890.4 [650.2;1066.4]	0.11
V30Gy	554.2 [336.6;786.0]	576.8 [398.7;730.8]	0.21
V45Gy	313.9 [164.3;446.9]	341.4 [210.2;451.2]	0.41
**Bowel bag** (cm^3)			
V15Gy	598.2 [374.9;823.7]	599.0 [415.6;813.9]	0.31
V30Gy	362.6 [192.9;535.2]	365.5 [196.4;522.2]	0.44
V45Gy	157.1 [63.1;280.0]	172.3 [73.4;287.5]	0.80
**Bowel loops** (cm^3)			
V15Gy	437.6 [268.7;587.9]	411.1 [267.7;627.6]	0.63
V30Gy	267.5 [130.8;383.8]	211.1 [120.1;401.2]	0.99
V45Gy	124.1 [50.2;227.4]	117.7 [57.1;228.3]	0.77
**Terminal ileum 1** (cm^3)			
V10Gy	106.6 [21.1;165.3]	102.9 [8.1;163.7]	0.77
V15Gy	88.2 [9.0;151.7]	91.0 [4.3;151.5]	0.75
V20Gy	71.3 [3.1;134.7]	71.5 [1.2;128.3]	0.84
V25Gy	55.8 [0.9;115.2]	53.2[0.3;95.4]	0.92
V30Gy	36.1 [0;88.6]	41.8 [0;73.5]	0.80
V35Gy	24.2 [0;65.9]	25.7 [0;52.2]	0.73
V40Gy	17.0 [0;50.2]	16.6 [0;42.9]	0.75
**Terminal ileum 2** (cm^3)			
V10Gy	19.5 [0;52.0]	13 [0;60.9]	0.87
V15Gy	10.3 [0;48.4]	5.7 [0;57.7]	0.85
V20Gy	3.6 [0;36.1]	2.6 [0;43.4]	0.91
V25Gy	1.1 [0;28.4]	0.6 [0;27.5]	0.84

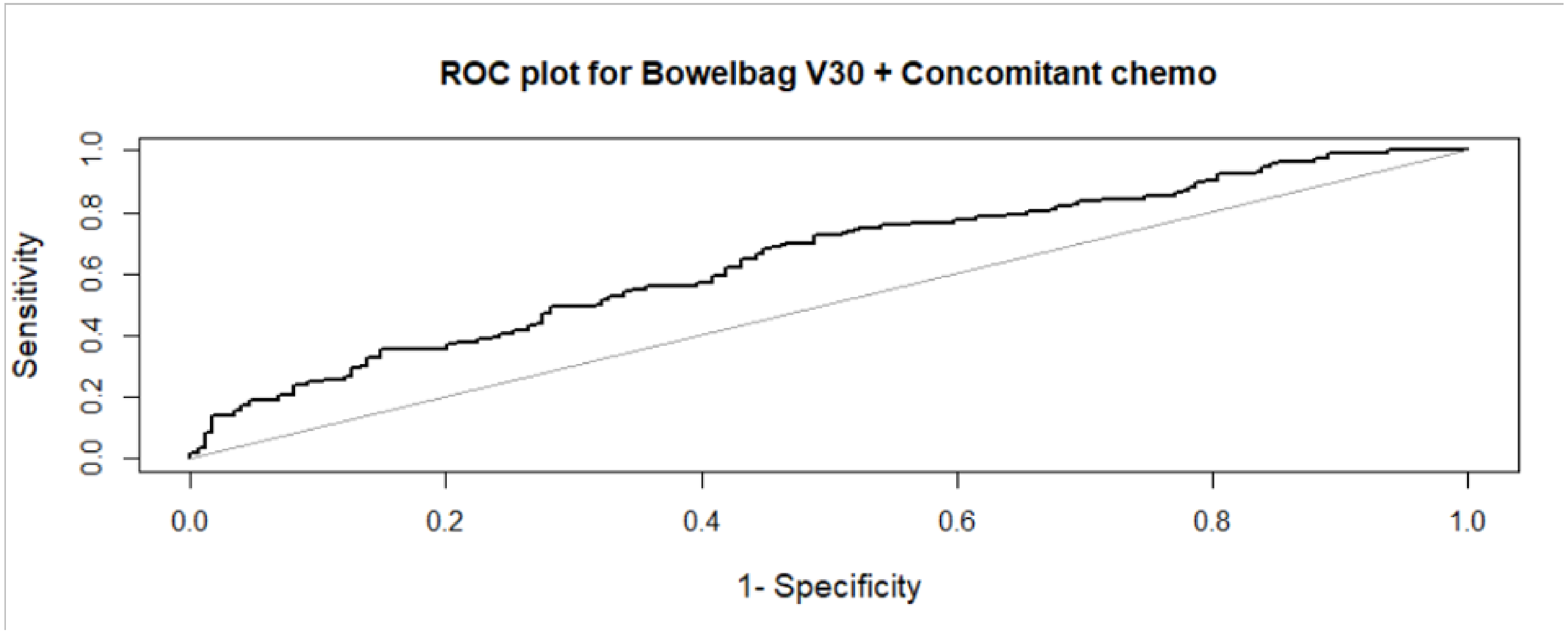

AUC [95% CI] for the NTCP model of Bowelbag V30: 0.646 [0.582 - 0.71]

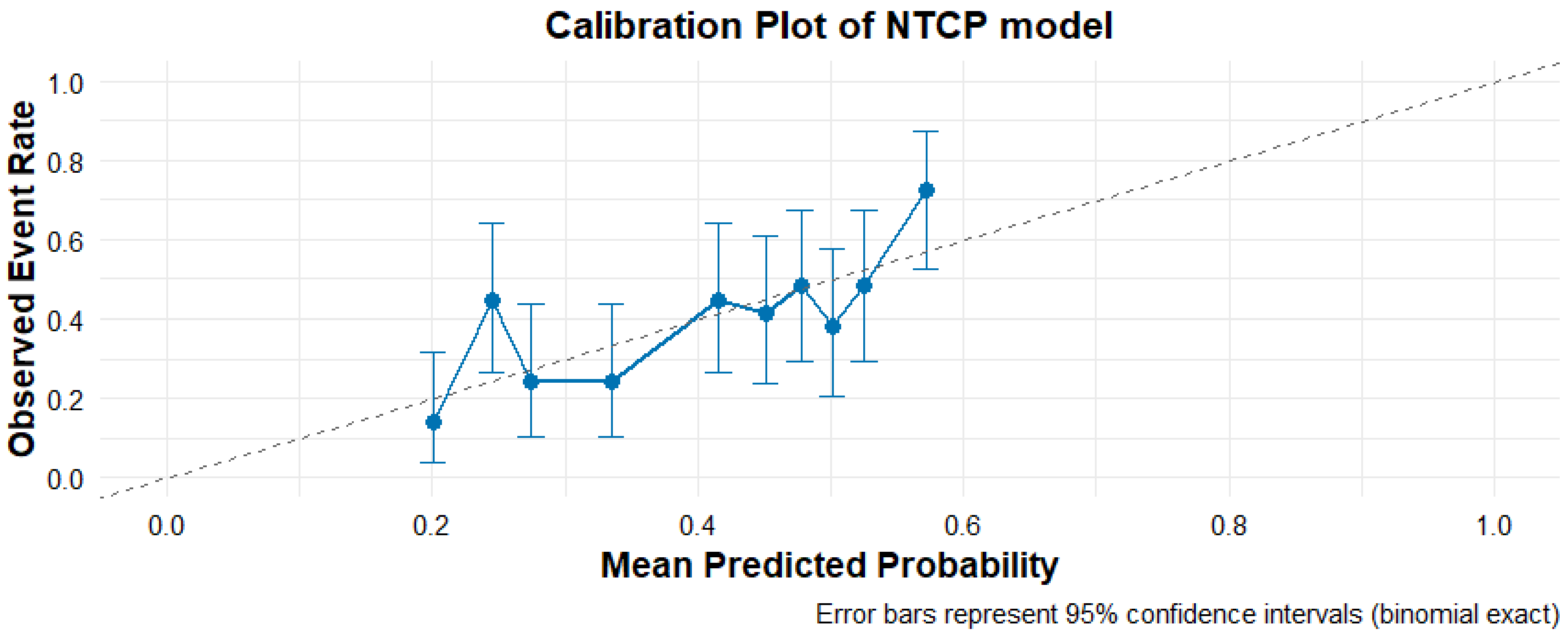

Calibration Plot of NTCP model
Observed Event Rate
1.0
0.8
0.6
0.4
0.2
0.0
0.0
0.2
0.4
0.6
0.8
1.0
Mean Predicted Probability
Error bars represent 95% confidence intervals (binomial exact)

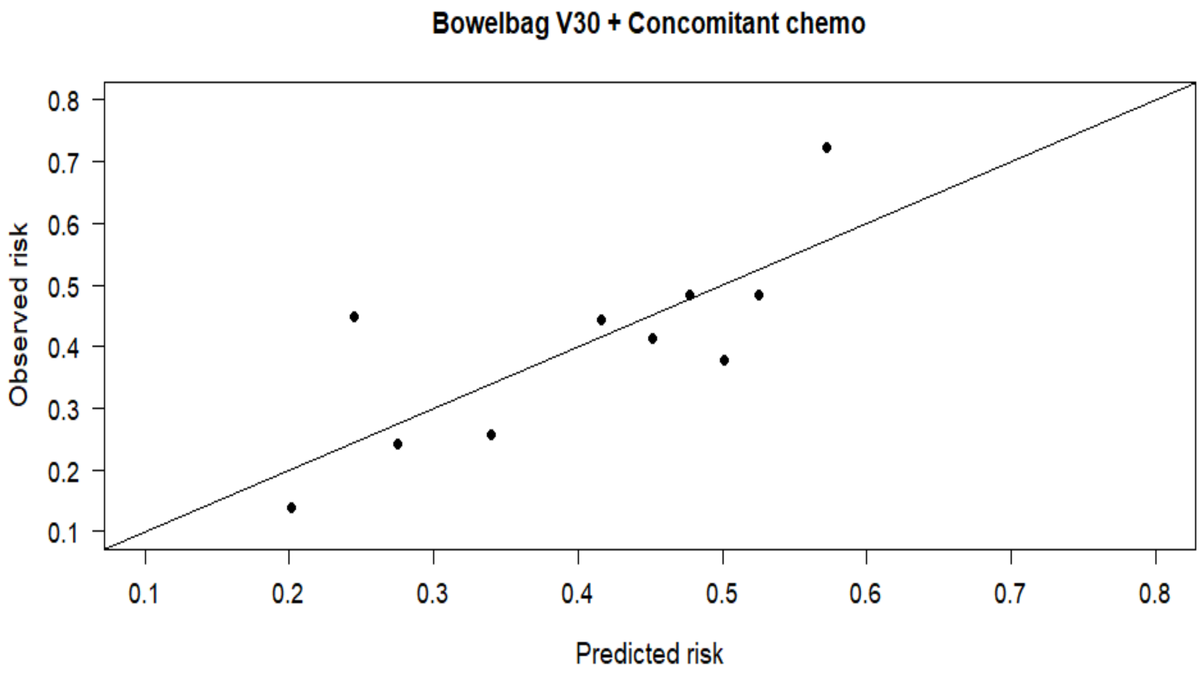

Goodnes-of-fit model of the NTCP model for bowelbag$_{V30Gy}$ and chemotherapy regimen, plotting predicted vs observed risk. P-value=0.1056.
